# Growth of *Ehrlichia canis*, the causative agent of canine monocytic ehrlichiosis, in vector and non-vector ixodid tick cell lines

**DOI:** 10.1016/j.ttbdis.2016.01.013

**Published:** 2016-06

**Authors:** Joana Ferrolho, Jennifer Simpson, Philippa Hawes, Erich Zweygarth, Lesley Bell-Sakyi

**Affiliations:** a The Pirbright Institute, Ash Road, Pirbright, Woking, Surrey, GU24 0NF, UK; b Institute for Comparative Tropical Medicine and Parasitology, Ludwig-Maximilians-Universität (LMU) München, Germany; c Department of Veterinary Tropical Diseases, Faculty of Veterinary Science, University of Pretoria, Onderstepoort 0110, South Africa

**Keywords:** *Ehrlichia canis*, *Rhipicephalus sanguineus* s.l., *Dermacentor variabilis*, Tick cell lines, *Ehrlichia ruminantium*, Electron microscopy

## Abstract

Canine monocytic ehrlichiosis is caused by *Ehrlichia canis*, a small gram-negative coccoid bacterium that infects circulating monocytes. The disease is transmitted by the brown dog tick *Rhipicephalus sanguineus* s.l. and is acknowledged as an important infectious disease of dogs and other members of the family Canidae worldwide. *E. canis* is routinely cultured *in vitro* in the canine monocyte-macrophage cell line DH82 and in non-vector *Ixodes scapularis* tick cell lines, but not in cells derived from its natural vector. Here we report infection and limited propagation of *E. canis* in the tick cell line RSE8 derived from the vector *R. sanguineus* s.l., and successful propagation through six passages in a cell line derived from the experimental vector *Dermacentor variabilis*. In addition, using bacteria semi-purified from *I. scapularis* cells we attempted to infect a panel of cell lines derived from non-vector species of the tick genera *Amblyomma*, *Dermacentor*, *Hyalomma*, *Ixodes* and *Rhipicephalus* with *E. canis* and, for comparison, the closely-related *Ehrlichia ruminantium*, causative agent of heartwater in ruminants. *Amblyomma* and non-vector *Dermacentor* spp. cell lines appeared refractory to infection with *E. canis* but supported growth of *E. ruminantium*, while some, but not all, cell lines derived from *Hyalomma*, *Ixodes* and *Rhipicephalus* spp. ticks supported growth of both pathogens. We also illustrated and compared the ultrastructural morphology of *E. canis* in DH82, RSE8 and *I. scapularis* IDE8 cells. This study confirms that *E. canis*, like *E. ruminantium*, is able to grow not only in cell lines derived from natural and experimental tick vectors but also in a wide range of other cell lines derived from tick species not known to transmit this pathogen.

## Introduction

Canine monocytic ehrlichiosis (CME) is a serious and sometimes fatal tick-borne disease of members of the family Canidae, predominantly dogs (Ewing, 1969, Skotarczak, 2003). The aetiological agent is the gram-negative obligate intracellular rickettsia *Ehrlichia canis* (family Anaplasmataceae, order Rickettsiales) (Dumler et al., 2001) that invades and develops in canine monocytes and macrophages, eventually leading to fever, depression, leucopaenia, thrombocytopaenia and death. The primary biological vector of *E. canis* is the brown dog tick *Rhipicephalus sanguineus* s.l. (Ewing, 1969, Groves et al., 1975, Harvey et al., 1979); experimental transmission of *E. canis* by the American dog tick *Dermacentor variabilis* has been reported (Johnson et al., 1998), while the argasid tick *Otobius megnini* failed to transmit the pathogen (Ewing et al., 1990).

Short-term cultivation of *E. canis* in monocyte cell cultures derived from dogs in the acute phase of the disease was reported over 40 years ago (Nyindo et al., 1971). Later, cells of a dog suffering from malignant histiocytosis gave rise to the continuous macrophage-monocyte cell line DH82 (Wellman et al., 1988) which was then used to continuously propagate *E. canis in vitro* at 37 °C (Dawson et al., 1991). In a study conducted by Ewing et al. (1995), in which several tick cell lines including one derived from *R. sanguineus* s.l. were inoculated with *E. canis*- infected leucocytes from infected dogs, it was only possible to successfully isolate and propagate the bacteria in the non-vector *Ixodes scapularis* cell line IDE8 (Munderloh et al., 1994). Subsequently a North American strain of *E. canis* was cultivated in another *I. scapularis* cell line, ISE6 (Singu et al., 2006), a South African strain was grown in a non-vector *Ixodes ricinus* cell line (Bell-Sakyi et al., 2007) and most recently Zweygarth et al. (2014) reported isolation and propagation of two South African and one Spanish strains of *E. canis* in IDE8 cells.

Although the continuous tick cell line RSE8 was established from embryonic *R. sanguineus* s.l. over 30 years ago (Kurtti et al., 1982), there has been no report of successful cultivation of *E. canis* in this or any other cell line derived from its natural vector. Two other ehrlichial species have been propagated in cell lines derived from their natural tick vectors: several geographically and antigenically distinct strains of *Ehrlichia ruminantium*, the causative agent of heartwater or cowdriosis of domestic ruminants, grow in two cell lines derived from the vector tick species *Amblyomma variegatum* (Bell-Sakyi, 2004), and the Arkansas strain of *Ehrlichia chaffeensis*, causative agent of human monocytic ehrlichiosis, grows in the *Amblyomma americanum* cell line AAE2 (Singu et al., 2006).

In this paper we report the results of attempts to propagate a Spanish strain of *E. canis* in a panel of 23 cell lines derived from the natural vector *R. sanguineus* s.l., the experimental vector *D. variabilis*, and 11 tick species of the ixodid genera *Amblyomma*, *Dermacentor*, *Hyalomma*, *Ixodes* and *Rhipicephalus* not known to transmit this pathogen*.* We compared the susceptibility of many of the tick cell lines to infection with *E. canis* and its close relative *E. ruminantium*, and also examined the ultrastructure of *E. canis* cultivated in tick and mammalian cells.

## Materials and methods

### Tick cell lines

Twenty-three cell lines derived from embryonic, moulting larval or moulting nymphal ticks of twelve ixodid tick species were tested for their ability to support growth of *E. canis* (Table 1). Uninfected cells were maintained in 2.2 ml volumes of complete L-15, H-Lac, L-15/MEM (Bell-Sakyi, 1991), L-15B (Munderloh and Kurtti, 1989) or L-15B300 (Munderloh et al., 1999) media or combinations thereof in sealed, flat-sided culture tubes (Nunc) in ambient air at 28 °C or 32 °C. Medium was changed weekly and subcultures carried out as required. Prior to infection with *E. canis*, the maintenance medium was removed and replaced with medium without antibiotics.

### *Ehrlichia canis* cultivation

*E. canis* (Spain 105) was isolated from blood of a naturally-infected dog into the *I. scapularis* cell line IDE8, and subsequently transferred into canine DH82 cells (Zweygarth et al., 2014). *E. canis*-infected DH82 and IDE8 cells were maintained in sealed 25 cm^2^ flasks at 32 °C in ambient air in 5 ml L-15B medium supplemented with 10% tryptose phosphate broth, 5% heat-inactivated foetal bovine serum, 0.1% bovine lipoprotein concentrate (MP Biomedicals), 0.1% NaHCO_3_ and 10 mM HEPES but without antibiotics (ECM) (Zweygarth et al., 2014) with weekly medium changes. *E. canis*-infected IDE8 cells were also grown continuously in sealed flat-sided culture tubes in 2.2 ml complete L-15B medium without antibiotics.

*E. canis* growth was monitored at 1–3 week intervals by microscopic examination of Giemsa-stained cytocentrifuge smears. Briefly, cells were resuspended and 50 μl aliquots of cell suspension were centrifuged for 5 min at 1000 x *g* (Shandon Cytospin 2) and air-dried. The resultant smears were fixed in technical methanol for 3 min and stained in 10% Giemsa for 20 min (Shute, 1966), rinsed twice with water buffered to pH 7.2 and air-dried. Stained smears were examined for presence of infection using a Leitz Orthoplan microscope at x 1000 magnification with oil immersion. *E. canis*-infected tick cell cultures were cryopreserved with 10% dimethyl sulphoxide in the vapour phase of a liquid nitrogen refrigerator as described previously (Bell-Sakyi, 2004).

### *Ehrlichia ruminantium* cultivation

*E. ruminantium* (Ball 3) (Haig, 1952) was maintained in *I. scapularis* IDE8 and ISE6 cells in flat-sided tubes or 25 cm^2^ flasks at 32 °C as described previously (Bell-Sakyi et al., 2000, Bell-Sakyi, 2004, Moniuszko et al., 2014). Infected cultures were monitored by cytocentrifuge smear as described above.

### Bacterial semi-purification and infection of tick cell cultures

Between 5 × 10^6^ and 1 × 10^7^ IDE8 or ISE6 cells infected with *E. canis* or *E. ruminantium* at a rate >50% were harvested by pipetting, 2–5 ml cell suspension was centrifuged at room temperature for 5 min at 200 x g, the supernatant was discarded and the cell pellet was resuspended in 500 μl of trypsin (500 μg/ml in PBS) and incubated for 20 min at 37 °C. The original volume was restored by adding ECM (*E. canis*) or L-15B (*E. ruminantium*) medium and the cell suspension was passed 10 times through a bent 26G needle to mechanically rupture the cells and release the intracellular bacteria. The resultant suspension was centrifuged at room temperature for 5 min at 1500 x *g*. Supernatant containing cell-free bacteria was collected and 200–500 μl aliquots were added to uninfected cell cultures growing in flat-sided tubes in ECM (*E. canis*) or complete L-15B (*E. ruminantium*). Uninfected cells were also inoculated on some occasions with 200–500 μl aliquots of supernatant from *Ehrlichia*-infected cell cultures centrifuged for 5 min at 1500 x *g* without prior digestion and disruption. Following inoculation, cultures were maintained in ECM (*E. canis*) or complete L-15B (*E. ruminantium*) and monitored for bacterial infection as above for up to 10 weeks, at which point if no infection was detected, the cultures were discarded. When *E. canis* cultures became heavily infected and began to destroy the host cells, subcultures were carried out if required onto fresh cells of the same line in ECM by transfer of 0.3–0.5 ml supernatant.

### Molecular confirmation of *E. canis* infection

DNA was extracted from uninfected and *E. canis*-infected RSE8 cell cultures using the DNeasy^®^ Blood and Tissue Kit (Qiagen) according to the protocol for purification of total DNA from animal blood or cells (Spin-Column protocol). A PCR was conducted using species-specific primers ECAN5 and HE3 (Murphy et al., 1998); a 20 μl reaction was prepared with 5 μl of 10x PCR buffer with MgCl_2_ (Promega), 1 μl of each primer at 10 μM, 1 μl of 10 mM dNTP Mix, 0.4 μl of Taq, 1 μl of DNA and nuclease-free water to make up to the final volume. The PCR was carried out with a thermal cycling profile of 95 °C for 1 min, and 35 cycles of 95 °C for 15 s, 55 °C for 15 s and 72 °C for 30 s, followed by a 72 °C extension for 7 min and a 4 °C hold (Veriti^®^ Thermal Cycler–Applied Biosystems). The PCR products were visualised by agarose gel electrophoresis.

### Transmission electron microscopy

Uninfected and *E. canis*-infected tick and DH82 cells were harvested as above, centrifuged for 5 min at 200 x g, washed once in PBS and resuspended in cold 2% glutaraldehyde in phosphate buffer. The cell suspensions were transferred to 1.5 ml Eppendorf tubes and immediately centrifuged to form a pellet. After 60 min the fixative was carefully removed, replaced with 1% aqueous osmium tetroxide and left at room temperature for a further 60 min. After dehydration in a graded series of ethanols (70% for 30 min, 90% for 15 min, 3 × 100% for 15 min each wash) the pellet was washed for 10 min in propylene oxide before infiltration with epoxy resin. Cell pellets were washed in a 1:1 mixture of propylene oxide and Agar 100 hard epoxy resin (Elektron Technology, Cambridge, UK) for 1 h before being washed in 100% Agar 100 resin for 2 h on a rotator. This resin was replaced with fresh resin before being polymerised at 60 °C for 18 h, after which the Eppendorf tube was cut away from the polymerised resin block. Ultra-thin sections (70 nm) were cut using a Leica UC6 ultramicrotome, stained with uranyl acetate and lead citrate using a Leica AC20 staining machine and imaged at 100 kV in a FEI T12 transmission electron microscopy using a Tietz F214 CCD camera.

## Results

**Fig. 1:**
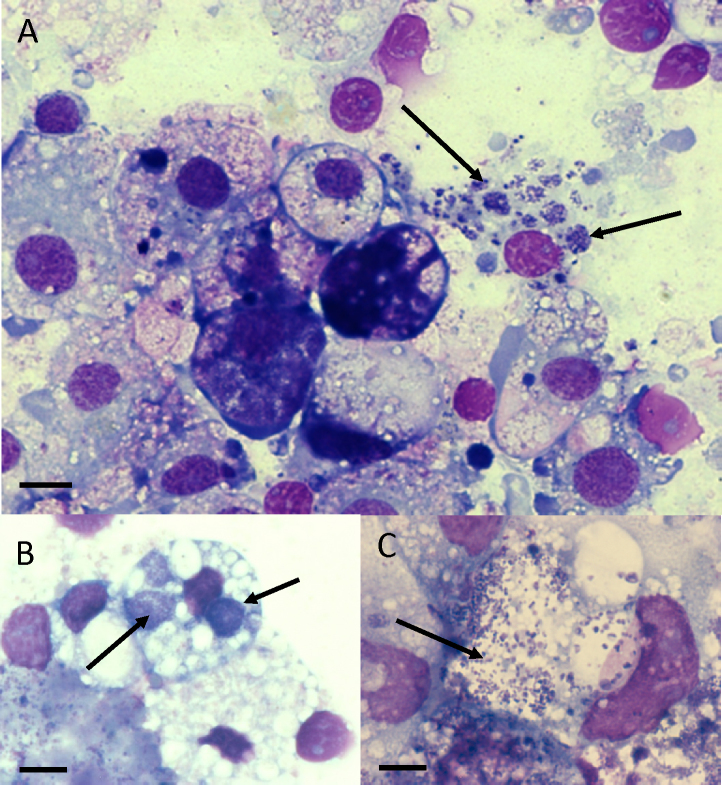
Infection of tick cell lines with *Ehrlichia canis*. A: RSE8 cells 14 days post inoculation. B: DVE1 cells at passage 4, 154 days post original inoculation. C: HAE/CTVM8 cells 91 days post inoculation. Cytocentrifuge smears of resuspended cells stained with Giemsa; images taken using a Zeiss AxioSkop 2 Plus microscope and Zeiss Axiovision software; x100 oil immersion objective; arrows indicate *E. canis* morulae; scale bars = 10 μm.

**Fig. 2:**
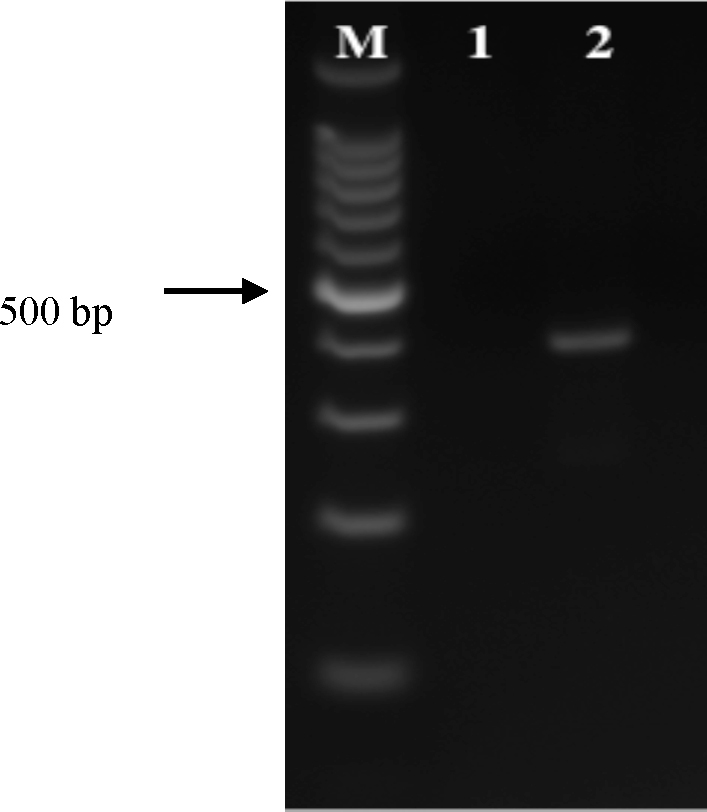
PCR amplification of a fragment of the *Ehrlichia canis* 16S rRNA gene from infected RSE8 cell DNA. Lane M: 100 bp marker; Lane 1: Negative control DNA from uninfected RSE8 cells; Lane 2: DNA from *E. canis*-infected RSE8 cells showing 396 bp product of *E. canis* 16S rRNA gene PCR-amplified using species-specific primers ECAN5/HE3. Arrow indicates 500 bp.

The cell line RSE8, derived from the natural vector *R. sanguineus* s.l., was successfully infected with *E. canis* semi-purified from IDE8 cells on 3/6 occasions (Table 1). Intracellular *E. canis* morulae were first seen in RSE8 cultures on day 14 post inoculation (Fig. 1A); no bacteria were seen in uninfected control cultures. Infection was maintained for 4 weeks but attempts to subculture the bacteria onto fresh RSE8 cultures were unsuccessful; aliquots were cryopreserved at day 17 post inoculation. Presence of *E. canis* in the infected RSE8 cultures was confirmed by PCR amplification of a 396 bp fragment of the 16S rRNA gene while no PCR product was amplified from uninfected cells (Fig. 2). Three attempts to infect the *R. sanguineus* s.l. cell line RML-RSE with *E. canis* harvested from IDE8 culture supernate failed (Table 1); on each occasion other aliquots of the same supernate successfully infected at least one other tick cell line. IDE8-derived *E. canis* also successfully infected the cell line DVE1 derived from the experimental vector *D. variabilis* (Fig. 1B); the infection was maintained in DVE1 cells through six passages over a period of 227 days, after which aliquots of the cultures were cryopreserved.

Of the cell lines derived from non-vector tick species, *E. canis* grew almost as well in the *I. scapularis* lines IDE2 and ISE18 as in IDE8 cells, and also established a low-level infection in the *I. ricinus* line IRE11, but failed to infect the *I. ricinus* lines IRE/CTVM19 and IRE/CTVM20. *E. canis* grew in all four *Rhipicephalus appendiculatus* and two *Rhipicephalus evertsi* lines tested, and in one cell line each derived from *Hyalomma anatolicum* (Fig. 1 C) and *Rhipicephalus* (*Boophilus*) *microplus*, with infection rates ranging from <1% to >50% (Table 1). Cell lines derived from the two *Amblyomma* spp. and the three non-vector *Dermacentor* spp. appeared refractory to *E. canis* infection.

Amongst the tick species from which cell lines were examined, *E. canis* appeared to have a slightly narrower spectrum of susceptibility than *E. ruminantium*. In addition to those lines previously tested for ability to support growth of the latter pathogen (Bell-Sakyi, 2004), cell lines derived from *Amblyomma americanum*, *Dermacentor andersoni*, *Dermacentor albipictus*, *Dermacentor nitens*, *R. sanguineus* s.l. and one previously untested line each derived from *R. appendiculatus* and *R.* (*B.*) *microplus* were all successfully infected with the Ball3 strain of *E. ruminantium* (Table 1). On the other hand, *E. canis* successfully infected a cell line derived from *H. anatolicum*, while *E. ruminantium* failed to infect another cell line derived from the same tick species (Bell-Sakyi, 2004).

**Fig. 3:**
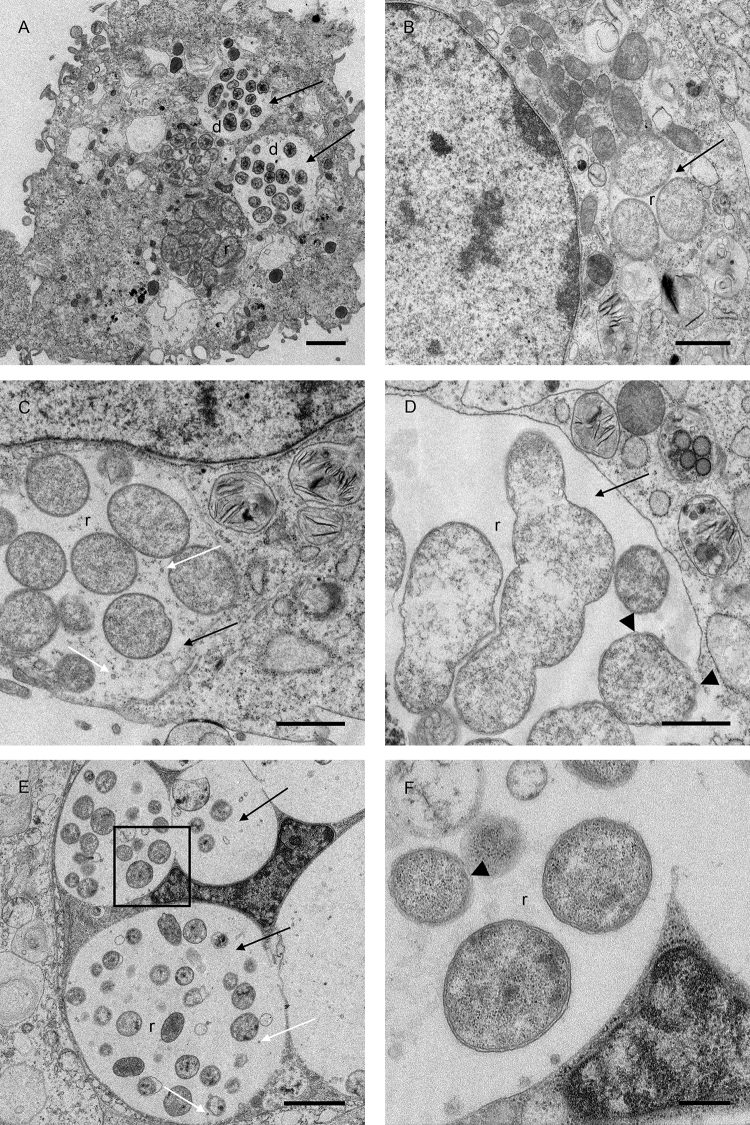
Transmission electron micrographs of *Ehrlichia canis*-infected cells. *E. canis* morulae (black arrows) in the cytoplasm of DH82 (A), IDE8 (B, C and D) and RSE8 (E and F) cells. Colonies containing reticulate (r) and dense-cored (d) forms are visible, as are small vesicles (white arrows in C and E) within the morular matrix. The area in the black box (E) is shown at higher magnification in F and clearly shows that the bacteria have a double membrane which in some cases appears to be slightly ruffled (arrowheads in D and F). Scale bars = 2 μm (A and E), 1 μm (B, C and D), 500 nm (F).

Transmission electron microscopy of *E. canis* in DH82 cells revealed morulae containing tightly-packed rounded, double membrane-bound reticulate forms or loosely-arranged, dense-cored forms often within the same host cell (Fig. 3A). Only reticulate forms were seen in infected IDE8 cells (Fig. 3B, C and D); these were generally more loosely-packed than in DH82 cells and rounded or pleomorphic. *E. canis* colonies seen in RSE8 cells (Fig. 3E and F) were generally larger than those in the DH82 and IDE8 cells with more pleomorphic and loosely-arranged bacteria; again, both reticulate (Fig. 3E) and dense-cored (data not shown) organisms were present. Bacteria with slightly ruffled membranes were seen in IDE8 (Fig. 3D) and RSE8 (Fig. 3F) cells. Small, pleomorphic vesicles were present in the matrix surrounding the bacteria within the morulae in IDE8 (Fig. 3C) and RSE8 (Fig. 3E) cells but not seen in morulae in DH82 cells.

## Discussion

The present study reports for the first time successful infection with *E. canis* of a cell line, RSE8, derived from its natural vector, using as inoculum bacteria isolated from infected IDE8 cells. Since the failure by Ewing et al. (1995) to isolate *E. canis* in an unspecified cell line derived from *R. sanguineus* s.l., no further attempts to propagate this pathogen in any vector-derived cell line have been reported. Establishing an infection in a cell line derived from the natural vector tick would open new opportunities for research to expand knowledge of the *E. canis* life cycle in the tick. As well as helping to understand the interaction between the bacterium and the tick at the cellular and molecular level, such research might ultimately lead to new strategies for CME control and prevention.

In contrast to the continuous cultivation of *E. canis* (Spain 105) achieved in IDE8 cells (Zweygarth et al., 2014), it was not possible in the present study to maintain infection of RSE8 cells beyond 4 weeks, and attempts to subculture the bacteria into fresh RSE8 cultures were unsuccessful. Moreover, a second *R. sanguineus* s.l. cell line, RML-RSE, proved completely refractory to infection with *E. canis* (Spain 105), suggesting the possibility that not all *R. sanguineus* s.l. populations are competent vectors of this pathogen as recently postulated (Cicuttin et al., 2015). While the RSE8 and RML-RSE cell lines were both established in laboratories in the USA, and therefore it is likely that the parent ticks belonged to North American populations, their exact geographic origin was not reported (Kurtti et al., 1982, Yunker et al., 1984). Genetic differences identified between populations of ticks historically identified as *R. sanguineus* on morphological grounds (Moraes-Filho et al., 2011) may be sufficient to affect the ability of cell lines derived from different parent ticks to support *E. canis* infection *in vitro*.

On the other hand, the single cell line derived from the experimental vector *D. variabilis*, DVE1, was highly susceptible to *E. canis* infection and supported continuous cultivation over five passages. A further ten previously untested cell lines, derived from six non-vector tick species, were found to support growth of *E. canis*, making a total of 15 tick cell lines permissive for one or more strains of the pathogen (Table 1). Eight previously untested tick cell lines were found to support growth of *E. ruminantium*; these included RSE8 and lines derived from *A. americanum* and three New World *Dermacentor* spp, increasing to five the number of ixodid tick genera whose cells are capable of *in vitro* infection with *E. ruminantium* (Bell-Sakyi, 2004).

While the ability of *E. canis* to infect and grow in *R. sanguineus* s.l. and *D. variabilis* cell lines correlates well with the known vector range of this pathogen, additional *Rhipicephalus* species such as *R. appendiculatus* and *R. evertsi*, whose cells support growth of *E. canis*, should be assessed for ability to transmit the pathogen as they occasionally infest dogs (Horak et al., 2009).

In previous studies, *E. canis* was propagated in tick cells at 34 °C (Ewing et al., 1995, Singu et al., 2006, Zweygarth et al., 2014), in medium with alkaline pH. In the present study, *E. canis* grew well in tick cell lines maintained at both 32 °C and 28 °C (Table 1) and in both ECM in which a high pH of ∼7.5 was maintained by addition of sodium bicarbonate and HEPES buffer, and complete L-15B in which the pH was always below 7.0. *E. ruminantium* also establishes and grows well in tick cells at acidic pH and at temperatures between 28 °C and 32 °C (Bell-Sakyi, 2004).

The ultrastructure of *E. canis* in DH82 cells was generally similar to that described by Popov et al. (1998), although the fibrils and tubular vesicles reported to occur in the intra-morular matrix surrounding the bacteria were not seen in the present study. The morphology of *E. canis* in IDE8 cells resembled that of *E. ruminantium* in the same cell line (Bell-Sakyi et al., 2000), although the electron-dense inclusion bodies seen within some *E. ruminantium* morulae were not observed with *E. canis*. Electron micrographs of *Ehrlichia mineirensis* in IDE8 cells (Cabezas-Cruz et al., 2013) revealed the same general pattern of morulae containing rounded or pleomorphic bacteria but with different texture to *E. canis* and *E. ruminantium*; this could be explained by the different sample processing protocol (high pressure freezing and freeze-substitution) used for *E. mineirensis*. Small vesicles seen in the intra-morular matrix of *E. canis*-infected cells were also reported for tick cells infected with *E. ruminantium* (Bell-Sakyi et al., 2000), *E. chaffeensis* (Dedonder et al., 2012) and *E. mineirensis* (Cabezas-Cruz et al., 2013), while ruffled outer bacterial membranes were described in *E. chaffeensis* and *E. mineirensis* but not *E. ruminantium*.

Of the four *Ehrlichia* spp. that can be propagated *in vitro* in tick cells, *E. canis*, *E. ruminantium*, *E. chaffeensis* and *E. mineirensis*, all grow well in one or more cell lines derived from the non-vector tick *I. scapularis*. Three of the four also grow in cell lines derived from their natural vectors (Bell-Sakyi et al., 2000, Singu et al., 2006), while there has been no report of attempted propagation of *E. mineirensis* in cell lines derived from *R. microplus*, the tick species from which it was originally isolated (Cabezas-Cruz et al., 2012). In the present study there was some overlap in the spectrum of tick cell lines supporting growth of *E. canis* and *E. ruminantium*; it would be interesting to test the same cell lines for ability to support growth of *E. chaffeensis* and *E. mineirensis*. Such studies might help to elucidate the factors governing *in vivo* vector competence of different ixodid tick species for these closely-related bacteria.

## Figures and Tables

**Table 1:** Tick cell lines tested for ability to support growth of *Ehrlichia canis* and *Ehrlichia ruminantium* in the present study and previously reported studies. The origins of the tick cell lines are cited by Alberdi et al. (2012) and Bell-Sakyi et al. (2015). *Ehrlichia* growth was monitored in Giemsa-stained cytocentrifuge smears: + = <1% of cells infected; ++ = 1–50% cells infected; +++ = >50% cells infected; − = no infected cells seen; ND = not done.

Tick species	Cell line	Incubation temperature	*E. canis* growth	*E. ruminantium* growth
*Amblyomma americanum*	AAE12	32 °C	−	++
*Amblyomma variegatum*	AVL/CTVM13	32 °C	−	++ +^d^
*Dermacentor andersoni*	DAE15	32 °C	−	++
	DAE100 T	32 °C	−	++
*Dermacentor albipictus*	DALBE3	32 °C	−	++
*Dermacentor nitens*	ANE58	32 °C	−	++
*Dermacentor variabilis*	DVE1	32 °C	+++	ND
*Hyalomma anatolicum*	HAE/CTVM8	32 °C	++	ND
*Ixodes ricinus*	IRE/CTVM18	28 °C	+^a^	+^d^
	IRE/CTVM19	28 °C	−	ND
	IRE/CTVM20	28 °C	−	ND
	IRE11	32 °C	+	ND
*Ixodes scapularis*	IDE2	32 °C	++	ND
	IDE8	32-34 °C	++ +^b^	++ +^d^
	ISE6	32-34 °C	++ +^c^	++^e^
	ISE18	32 °C	++	ND
*Rhipicephalus appendiculatus*	RAE/CTVM1	32 °C	+	++ +^d^
	RAN/CTVM3	28 °C	++	++ +^d^
	RAE25	32 °C	+++	++ +^d^
	RA243	32 °C	−	++
*Rhipicephalus evertsi*	REE/CTVM31	28 °C	++	ND
	REN/CTVM32	28 °C	++	ND
*Rhipicephalus sanguineus*	RSE8	32 °C	+	+
	RML-RSE	28 °C	−	ND
*Rhipicephalus* (*Boophilus*) *microplus*	BME/CTVM23	32 °C	+	++

a Bell-Sakyi et al. (2007)

b Ewing et al., 1995, Zweygarth et al., 2014

c Singu et al. (2006)

d Bell-Sakyi (2004)

e Moniuszko et al. (2014)
